# Development and Evaluation of an Accelerometer-Based Protocol for Measuring Physical Activity Levels in Cancer Survivors: Development and Usability Study

**DOI:** 10.2196/18491

**Published:** 2020-09-24

**Authors:** Tracy E Crane, Meghan B Skiba, Austin Miller, David O Garcia, Cynthia A Thomson

**Affiliations:** 1 Department of Biobehavioral Health Sciences College of Nursing University of Arizona Tucson, AZ United States; 2 Department of Health Promotion Sciences Mel and Enid Zuckerman College of Public Health University of Arizona Tucson, AZ United States; 3 Department of Biostatistics Roswell Park Cancer Institute Buffalo, NY United States

**Keywords:** wearable electronic devices, physical activity, cancer survivors, activity trackers, mobile phone

## Abstract

**Background:**

The collection of self-reported physical activity using validated questionnaires has known bias and measurement error.

**Objective:**

Accelerometry, an objective measure of daily activity, increases the rigor and accuracy of physical activity measurements. Here, we describe the methodology and related protocols for accelerometry data collection and quality assurance using the Actigraph GT9X accelerometer data collection in a convenience sample of ovarian cancer survivors enrolled in GOG/NRG 0225, a 24-month randomized controlled trial of diet and physical activity intervention versus attention control.

**Methods:**

From July 2015 to December 2019, accelerometers were mailed on 1337 separate occasions to 580 study participants to wear at 4 time points (baseline, 6, 12, and 24 months) for 7 consecutive days. Study staff contacted participants via telephone to confirm their availability to wear the accelerometers and reviewed instructions and procedures regarding the return of the accelerometers and assisted with any technology concerns.

**Results:**

We evaluated factors associated with wear compliance, including activity tracking, use of a mobile app, and demographic characteristics with chi-square tests and logistic regression. Compliant data, defined as ≥4 consecutive days with ≥10 hours daily wear time, exceeded 90% at all study time points. Activity tracking, but no other characteristics, was significantly associated with compliant data at all time points (*P*<.001). This implementation of data collection through accelerometry provided highly compliant and usable activity data in women who recently completed treatment for ovarian cancer.

**Conclusions:**

The high compliance and data quality associated with this protocol suggest that it could be disseminated to support researchers who seek to collect robust objective activity data in cancer survivors residing in a wide geographic area.

## Introduction

### Background

For cancer survivors, it has been demonstrated that physical activity has positive effects on psychosocial and physical outcomes, including weight management, quality of life, fatigue, emotional well-being, and sleep as well as social, cognitive, and physical functioning [1-3]. However, self-reported levels of physical activity among ovarian cancer survivors are low, with approximately 20% of women meeting the recommended 150 min of moderate-to-vigorous physical activity per week [4,5]. Measurement and assessment of physical activity in cancer survivors remain a challenge in randomized controlled trials, wherein self-reported questionnaires are most commonly used [6]. Subjective self-report of physical activity is wrought with significant recall and measurement error bias, as many study participants overreport moderate- and vigorous-intensity physical activities [7]. In previous trials, participants not only overreported physical activity but also there was a concurrent tendency to underreport sedentary time [8]. Furthermore, many self-report instruments fall short in capturing light-intensity physical activity [9], a major source of activity in adults, particularly cancer survivors [10]. In fact, in the Women’s Health Study, fewer than 50% of women met physical activity guidelines as measured using accelerometry compared with approximately 67% women from self-reported physical activity [11]. Objective measurement of activity is, therefore, considered the gold standard for assessing physical activity exposure for all levels of intensity (rest, sedentary, light intensity, and moderate-to-vigorous intensity) in clinical trials and epidemiological studies.

Accelerometry provides the opportunity to objectively evaluate the minutes of activity per day, intensity, and total energy expenditure of physical activity as well as sedentary time, known factors associated with cancer risk [12,13]. Accelerometer protocols have been successfully implemented in population-level surveillance studies [10]. However, the existing information related to distance accelerometer methods (eg, mail based) has suggested poor compliance, with minimal improvement in recent years despite the growing use of this technique in assessing physical activity [14]. The use of accelerometry to capture objective activity data is particularly scarce among randomized and specifically lifestyle intervention trials in cancer survivors. A recent review highlighted that methods of accelerometry data collection among cancer survivors varied and were inconsistent, with limited studies reporting necessary details regarding data collection, compliance, and processing of data that would support replication of the research [15]. Robust measurement of physical activity using objective methods in cancer survivors is of high importance. Cancer survivors have unique needs as a result of cancer and subsequent treatment, including but not limited to fatigue, ostomies, abdominal pain, and chemotherapy-induced peripheral neuropathy [4,16,17], all of which can be a barrier to physical activity.

### Objectives

To improve the rigor of physical activity assessment in both epidemiologic and intervention studies, a standardized protocol for collecting reliable and valid mail-based accelerometer data is warranted. In this paper, we describe a mail-based protocol for prospective collection of accelerometry data in a convenience sample of 580 ovarian cancer survivors enrolled in a diet and physical activity intervention. Of note, study participants resided in 48 US states, suggesting that this protocol may be applicable and implemented in studies that recruit participants across wider geographic areas. Finally, the protocol was tested across multiple study time points to demonstrate compliance over time, a critical aspect of longitudinal research.

## Methods

### Wearable Electronic Device

The Actigraph GT9X Link is a validated triaxial accelerometer [18] that includes a gyroscope, magnetometer, secondary accelerometer, and Bluetooth capability [19] manufactured by ActiGraph, LLC. The Actigraph GT9X Link uses the same validated algorithms from its predecessor, GT3X, which was validated through indirect calorimetry [20]. Compared with GT3X, GT9X Link captures different step counts [21] but still provides comparable data and estimates of activity intensity and sedentary time [22]. Few studies have implemented GT9X Link to assess physical activity to date, but none have been in cancer survivors [23-25].

Physical activity was measured within the implementation of the GOG/NRG 0225 Lifestyle Intervention for oVarian cancer Enhanced Survival (LIvES) study (NCT00719303). Briefly, the LIvES study is a randomized controlled trial that tested a 24-month lifestyle intervention (high vegetable and fiber, low-fat diet with daily physical activity goals) compared with an attention control (general health education) on ovarian cancer progression-free survival. Eligible participants were in 6 weeks to 6 months postcancer treatment for stages II to IV disease and were randomized in a ratio of 1:1 to intervention versus control groups, stratified by receipt of consolidation therapy. Women were enrolled at NRG Oncology clinic sites nationwide. Intervention components are delivered via telephone by trained health coaches from the University of Arizona Cancer Center (UACC). Protocol and related methodologies as well as retention approaches for this study were previously published [26]. Measures included self-report physical activity assessment via the validated Arizona Physical Activity Questionnaire (APAQ) [27] and repeat accelerometer-measured activity at time points aligned with the self-report measure (baseline, 6 months, 12 months, and 24 months) as a measure of intervention adherence. The APAQ also included self-report of participants’ height and weight, which was used to calculate their BMI. Participant demographics were collected using a standardized form at enrollment.

### Accelerometer Protocol

**Figure 1:**
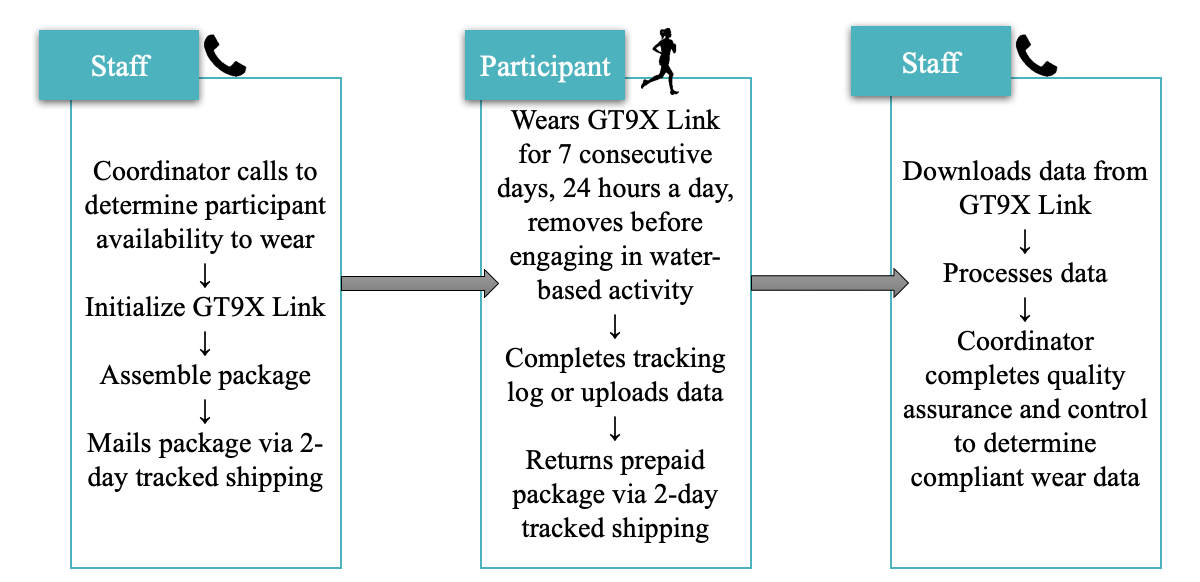
Participant communication schema for accelerometry data capture in the Lifestyle Intervention for oVarian cancer Enhanced Survival study.

The accelerometer protocol was developed using manufacturer instructions, a review of published literature [28-32], and prior experience of study investigators. The protocol development process included the creation of standardized instructional materials, a staff training program, implementation strategies, and data quality assessments. The objective activity was assessed using the GT9X Link accelerometer at 4 study time points: baseline, 6 months, 12 months, and 24 months (Figure 1). Participants were asked to wear GT9X Link on their nondominant hip for a continuous period of 7 days, 24 hours a day, with the exception of water-based activities such as showering or swimming.

### Participant Instructional Materials

The instructional materials for this protocol included a cover letter that summarized the procedures, guidance on wear, troubleshooting, and tracking logs for wear time. Before implementing the protocol, study staff and 2 individuals outside of the study (who were of the same age as study participants) beta-tested the materials to determine usability and acceptability of the instructional packet, tracking log, and mobile device app. Feedback from usability and acceptability testing was incorporated into the final study documents. The instruction packet was revised, as technology related to GT9X Link (such as chargers and waist clips) was updated in the inventory by ActiGraph.

An introductory letter was included in each package that oriented participants to the activity monitor portion of the study and acknowledged and thanked them for their study participation to date, specifically in relation to data collection at prior time points. The letter detailed the list of materials included in the mailed package and provided a study telephone number to call if they had any questions. The instructional booklet served as a resource for participants and included written instructions with photos that described how to (1) charge the activity monitor, (2) wear the activity monitor on the nondominant hip, (3) complete the tracking log, (4) download the CentrePoint Study Admin Sync app on a mobile device for data upload, and (5) prepare the package for return to the data collection center at the end of the 7-day wear period. At baseline, for participants who provided an email address, an instructional video that detailed the same information contained in the written instructional booklet was sent via email with the embedded web link. A monthly calendar was provided as a resource for participants to mark which days of the week the activity monitor was required to be worn and the date to return the accelerometer. Finally, a checklist of related Actigraph GT9X Link wear procedures was included as a tool to remind the participants of each step in the process and to promote inclusion of the activity log in the return package to the UACC Behavioral Measurement and Interventions Shared Resource (BMISR).

### Self-Monitoring

A tracking log was provided to all participants to self-monitor wear time and record all accelerometer wear time on a daily basis for the 7-day wear period. The tracking log detailed self-reported measures of wake and sleep times as well as removal times of the accelerometer and indicated if the data were uploaded via the CentrePoint Study Admin Sync app. These data were used as quality assurance measures to determine the correct wear times for individual participants (which were then verified by accelerometer readings) as well as compliance with self-monitoring behaviors. Engagement with the CentrePoint mobile app, defined as a mobile upload of data during the 7-day wear period at least once, was confirmed by the researcher through the backend of the CentrePoint mobile app.

### Staff Training

Study staff working with accelerometer data collection were trained and adhered to a standard operating procedure (SOP), specifically created for objective physical activity data collection in the context of the study. The SOP included guidance for staff in relation to initializing the accelerometer and downloading data from the Actigraph GT9X Link as well as the procedure for contacting participants (eg, phone script and number of call attempts) and mailing the packages. These SOPs were informed by previous studies that used the Actigraph GT9X Link accelerometers for the collection of physical activity data and further adapted for the study population in an effort to enhance compliance beyond prior reports [28-32].

### Mailing Approaches

After confirming the availability to wear the accelerometer, the study staff created packages to send to the participant’s home address. The packages included (1) introduction letter, (2) tracking log, (3) instruction packet, (4) charging pack, (5) return checklist, (6) monthly study activity calendar, and (7) return postage paid preaddressed return envelope. The United States Postal Service (USPS) Priority Flat Rate padded envelopes were used for sending and receiving packages. At the time of this study, USPS Priority mail provided 2-day shipping to the Continental United States, which was chosen to reduce the likelihood of an accelerometer losing charge in transit and expedite the wearing process to ensure that data could be collected in an appropriate time frame. USPS tracking numbers were assigned to each outgoing and incoming package and documented. Study staff would access updates of the location of the package through a web-based tool maintained by the USPS. The expected battery life of GT9X Link is 10 days; however, to ensure that battery life lasted through the duration of the shipping and wear period, a charging pack was included with the accelerometer. The window to open the accelerometer package and initiate wear time was ±2 weeks (14 days) at each time point. An extended window of an additional 4 weeks was used in extenuating circumstances such as extended travel, illness, or reissue of the accelerometer because of damage or loss. At the end of the wear duration, participants mailed the activity monitor, charging pack, and tracking log in the prepaid and addressed return envelope provided in the original package.

### Communication

At each study time point, participants were contacted by trained study staff from UACC-BMISR via telephone. During this call, the participant was queried as to when and whether they would be available to wear the accelerometer for 7 consecutive days. If they were agreeable to wearing the accelerometer through verbal consent and confirmation, a follow-up call occurred within 2 days of confirmed delivery of the package to determine receipt by the participant and provide a detailed explanation of wear and documentation procedures. Predetermined wear dates were estimated by the study staff, including setting a return date estimated and documented as the last day of anticipated wear. An additional 4 days were added for return shipping. Participants were instructed to wear the accelerometer continuously for 24 hours a day for a total duration of 7 days, including during sleep, with the exception of when bathing, showering, or swimming. Participants were instructed to first fully charge the activity monitor upon receipt before wearing (and if the battery life dropped to <10%) and to start the wear time upon awakening the next day and continue to wear through wake time after day 7. For the duration of wear time, participants were requested to complete a tracking log that included date, time awake, time asleep, times they took off the device, and the reason the device was removed. If 1 of the wear days was missed, they were asked to wear the device for an additional day and document on the tracking log reason for nonwear (eg, clinic appointment, illness, and forgot).

A few areas of protocol implementation required troubleshooting to enhance compliance. The first was to reach the participant for the initial accelerometer distribution. Following the SOP, the study staff attempted to contact a participant twice a week for 2 consecutive weeks, after which the study coordinator would contact the clinic to ensure that contact information was correct before continuing further attempts to contact the participant. Second, select participants who delayed in returning the accelerometer. If an accelerometer was not received within 7 days of the anticipated return date, the participant would be called by the study coordinator and, if required, a voicemail was left. The expected return date was then updated. If still it was not received within 7 days of this extended return date, the participant was contacted again by telephone, and an email reminder was sent. If still not returned by 3 weeks after the initial expected return date, the oncology clinic where the participant gave consent for trial participation was contacted to confirm whether the participant was still active in the study and request that the clinic discusses the return of the accelerometer with the study participant during their next scheduled clinic visit. In addition, the study staff would attempt to contact the participant to return the accelerometer once a week for the next 4 weeks. If the accelerometer was not successfully returned after 2 consecutive months of contact attempts, the participant was considered protocol noncompliant, and a USPS-certified letter from the study’s principal investigator was mailed to the participant’s residential address to request the return of materials and provided instructions on how to do so.

### Data Collection and Quality Assessment

#### Data Collection and Capture

The study staff tracked contact attempts with participants at each time point using an encrypted shared spreadsheet. This spreadsheet contained no personal identifying information, only the study participant ID numbers. Name, contact number, email, and mailing address were stored separately on the Health Insurance Portability and Accountability Act (HIPAA)–secure study platform and database [33]. The enrollment date of the participant was added to this spreadsheet and used to calculate the open windows for subsequent time points. It also included the initialization date of the accelerometer, mailing date, and tracking numbers of the packages as well as return date, download date, and whether or not compliant data were collected. Any special notes about the participant during the specified time point were documented on the spreadsheet. Of note, if a participant refused to wear or was unable to wear an accelerometer at one time point, this was documented, and they were still queried at subsequent time points if they remained active in the study. Refusal to wear was defined as the participant declining to wear the accelerometer for any reason (eg, not having available time, inconvenience, conflicts with religious holidays, or discomfort with wearing the accelerometer). Unable to wear was defined as participants expressing willingness to wear an accelerometer but not having physical capacity to wear during open windows (eg, surgery, hospitalization, illness or injury, or natural disaster). Participants were removed from being contacted at future study time points if the participant had reached a study endpoint (eg, disease progression, other diseases, lost to follow-up, or withdraw of participation) or was previously protocol noncompliant (eg, did not return the accelerometer after 2 consecutive months of contact attempts).

Data collection included the use of the Actigraph GT9X Link Study Admin Sync, a web-based study management platform for accelerometers. This platform permits participant connection through CentrePoint Study Admin Sync, a mobile app available on iOS and Android devices that gives participants the option to upload their accelerometer data in real time to a secure HIPAA–compliant cloud-based study database [34]. At the time of developing this protocol, there was no literature available regarding participant uptake of the use of this mobile app. Therefore, the integration of this platform was an opportunity to explore the use of a mobile app as a data collection tool to inform future research. Participants were asked whether they used a mobile device (eg, smartphone or tablet) and whether they would be willing to download the mobile app that connects the mobile device to the accelerometer through Bluetooth connectivity and upload the data through user response to the associated study database in the Actigraph GT9X Link Study Admin Sync. During the follow-up call, if the participant opted to use the mobile app, instructions for downloading and installing the app on their mobile device were covered in detail. To connect Actigraph GT9X Link to the app, a unique 5-digit code was given to the participant via telephone. Participants entered this code on their device within a 2-min time frame before the code expired. This mobile app was optional for the participant to upload their data at the end of each waking period.

In an attempt to rigorously collect objective physical activity data, provisions were made to reissue an accelerometer if the accelerometer was lost and data could be captured within the predesignated extended wear time frame window of 4 weeks. The same protocol for initializing and mailing the replacement accelerometer was followed.

All accelerometers and accessories were sanitized by the study staff upon return to UACC-BMISR. A separate spreadsheet was used by the study staff to track all accelerometers in a study-specific inventory. If a participant reported a problem with their accelerometer or charger, this was documented by the study staff and investigated upon return of the accelerometer. If the problem could not be resolved, the accelerometer or charging dock would be removed from the mailing rotation and replaced.

The accelerometers were initialized, and the data were downloaded using the Actigraph GT9X Link CentrePoint Study Admin System. Weight and age were entered for each participant during initialization, and the time zone of the participant was documented by the study staff. Participants were blinded to the physical activity feedback from the device; the screen was programmed to only display battery life, date, and time.

#### Data Quality Assessment

As part of quality control, the study staff visually inspected all processed data for compliance. Compliance with the accelerometer wear time was defined by the investigator group before study initiation. These cut-off points have been previously validated in adults; no specific cut-off points currently exist for cancer survivors [35]. Specifically, wear compliance was set at ≥4 days, with ≥10 hours of daily wear time [36,37]. Initial data from the accelerometer were downloaded and exported to ActiLife (version 6.12) software. Accelerometer data were processed using 60-second epochs and Freedson [38] cut-off points.

### Statistical Analysis

Descriptive data are reported as frequency, means, or medians. Potential predictors of compliant data, including demographic characteristics and self-monitoring behavior, at baseline were evaluated using logistic regression models. As the sample size decreased longitudinally from participants reaching a predetermined study endpoint, logistic regression models were not repeated for subsequent time points. Comparison of factors related to self-monitoring behaviors and compliant accelerometry data collected at each time point were conducted using the Pearson chi-square tests. All statistics were completed in Stata 16 (StataCorp LLC).

## Results

### Overview

From July 2015 to December 2019, 580 cancer survivors active in the study were contacted by the study staff at enrollment to initiate the accelerometer procedures, 12.9% (75/580) provided an email address, and the majority preferred to communicate via telephone. Over the 4 time points, a total of 1337 individual accelerometer mailings were completed. At baseline, 98.4% (571/580) women were available and willing to wear the accelerometer—95.2% (533/560) study participants were compliant with the wear protocol and had usable data, and 5% (27/560) had insufficient wear time. For the 3.5% (20/580) participants without the accelerometer wear time at baseline, reasons for nonparticipation included participants who refused to wear the accelerometer (n=3), unable to wear because of illness or natural disasters (n=8), and off-study before completing baseline assessments (n=9).

At subsequent time points, participants who demonstrated protocol noncompliance (eg, not returning the accelerometer at a previous time point) were not asked to collect follow-up accelerometer data. In addition, if a participant reached a study endpoint, no further measurements of activity were completed. At 6 months, 95.2% (394/414) of individual participants were sent accelerometers, resulting in the acquisition of compliant data from 90.9% (358/394) of the active sample. At 12 months, 260 active participants were sent accelerometers for repeat activity measurement, with 95.4% (248/260) of women providing compliant data. At 24 months, 123 eligible participants were sent accelerometers, resulting in 96.7% (119/123) of the active sample that provided compliant data. The details of the accelerometer data compliance by time points are outlined in Table 1.

Across all time points, 49 accelerometers required a reissue for participant data collection. The majority (32/49, 65%) of the reissued accelerometers were at baseline. Of these reissues, 25% (12/49) were because of depleted battery life upon arrival, 20% (10/49) for software malfunction, and 20% (10/49) were lost by either the participant or in transit. Removal from mailing rotation and replacement of inventory for accelerometers that encountered issues and nonfunctional charging docks were 16 and 67, respectively.

Participants engaged in the accelerometer protocol were representative of the overall Lifestyle Intervention for oVarian cancer Enhanced Survival study population in terms of demographic and clinical characteristics [26]. On average, participants were aged 60.1 (SD 9.3) years, and the majority were non-Hispanic college graduates with a normal BMI (Table 2). The results from logistic regression models of age, education, ethnicity, and BMI as well as self-monitoring behaviors indicated that tracking log completion was the greatest predictor of compliant data at baseline (Table 3). Odds ratios (ORs) were significantly higher (OR 54.71, 95% CI 17.05-175.59; *P*<.001) for having compliant data if a participant completed a corresponding tracking log compared with those who did not complete the tracking log during the 7-day wear period. Other factors, including age, education, ethnicity, BMI, and mobile app engagement, were not significantly associated with compliant data at baseline.

**Table 1:** Details of the accelerometer wear time compliance in 580 participants by time points on the Lifestyle Intervention for oVarian cancer Enhanced Survival accelerometer protocol.

Study sample^a^			Baseline^a^, n (%); N	6 months^a^, n (%); N	12 months^a^, n (%); N	24, months^a^, n (%); N
**Total** **active^b^** **sample**			571 (98.4); 580	414 (71.4); 580	284 (49.0); 580	154 (28.7); 580
	**Agree to wear^c^**		560 (98.1); 571	394 (95.2); 414	260 (91.5); 284	123 (79.9); 154
		Compliant data^d^	533 (95.2); 560	358 (90.9); 394	248 (95.4); 260	119 (96.7); 123
		Noncompliant data^d^	27 (5); 560	36 (9); 394	12 (5); 260	4 (3); 123
	Refused to wear^e^		3 (<1); 571	16 (4); 414	21 (7); 284	24 (16); 154
	Unable to wear^f^		8 (1); 571	4 (1); 414	3 (1); 284	5 (3); 154
**Total** **inactive^g^**			9 (2); 580	166 (28.6); 580	296 (51.0); 580	384 (71.6); 536
	Protocol noncompliant^h^		N/A^i^	14 (8); 166	25 (8); 296	27 (7); 384
	Study endpoint^j^		9 (100); 9	152 (91.6); 166	271 (91.6); 296	357 (93.0); 384

^a^Values may not add up to 100% because of rounding.

^b^Active participants included those still on study time point and eligible to wear an activity monitor at each time point. At 24 months, 44 women were not yet at the study time point and, therefore, were not included in the sample size.

^c^Agree to wear is defined as a participant who provided verbal consent and confirmation of availability to wear the accelerometer via phone.

^d^Compliant data were defined as ≥4 consecutive days, with ≥10 hours of daily wear time. Percent is calculated by the number of all participants who provided verbal consent and were available to wear the accelerometer. Participants who were not active in the study were not included in the denominator for data compliance.

^e^Refusal to wear is defined as a participant who declined to wear the accelerometer for any reason.

^f^Unable to wear is defined as a participant who expressed willingness to wear the accelerometer but did not have the physical capacity to wear during the open window.

^g^Inactive participants included those who were protocol noncompliant or had reached a study endpoint.

^h^Protocol noncompliant is defined as a participant who did not return the accelerometer after 2 consecutive months of contact attempts.

^i^N/A: not applicable.

^j^Participants who reached a study endpoint, defined as disease progression, other diseases, lost to follow-up, or withdraw of participation, were not asked to wear the accelerometer at any future time points.

**Table 2:** Baseline characteristics for women who were enrolled in the Lifestyle Intervention for oVarian cancer Enhanced Survival accelerometer protocol at baseline (n=580).

Characteristics		Values
Age at enrollment (years), mean (SD)		60.07 (9.3)
**Education**^a^, **n (%)**		
	High school or less	75 (14.0)
	Some college education	147 (27.4)
	College graduate	314 (58.6)
**Ethnicity**^a^, **n (%)**		
	Hispanic	34 (6.4)
	Non-Hispanic	500 (93.6)
**BMI class (kg/m**^2^)**, n (%)**		
	Normal (18.5-24.9)	211 (36.4)
	Overweight (25.0-29.9)	203 (35.0)
	Obese (≥30.0)	166 (28.6)

^a^Missing data <8%.

**Table 3:** Predictors of baseline compliant data and association with demographic and anthropometric characteristics (n=522).

Characteristics^a,b^		Odds ratio (95% CI)		*P* value	
Age		1.00 (0.95-1.05)		>.99	
**Education**					
	High school or less		1.0^c^		N/A^d^
	Some college education		3.27 (0.74-14.44)		.12
	College graduate		2.60 (0.68-9.92)		.16
**Ethnicity**					
	Non-Hispanic		1.0		N/A
	Hispanic		0.89 (0.15-5.25)		.90
**BMI class**					
	Normal		1.0		N/A
	Overweight		1.17 (0.33-4.13)		.80
	Obese		0.88 (0.25-3.07)		.84
Tracking log completion		54.71 (17.05-175.59)		<.001	
Mobile app engagement		2.79 (0.56-13.84)		.21	

^a^Using data available for participants who agreed to wear an accelerometer at baseline.

^b^Only participants with nonmissing values for all variables are included in the logistic regression model.

^c^These are all provided with the exception of the referent group, which would *not* have a CI—this is the group we are comparing against to determine the OR and CI.

^d^N/A: not applicable.

### Compliance With Tracking Logs and Mobile App Engagement (Self-Monitoring)

At baseline, 88.6% (496/560) of the tracking logs were returned. At subsequent time points, 83.0% (327/394) were returned at 6 months, 84.2% (219/260) were returned at 12 months, and 86.2% (106/123) were returned at 24 months. The most common reason reported for the removal of the Actigraph GT9X-Link on the tracking logs was shower or bath, aligned with the wear protocol. All other reasons for removal accounted for <10% of the reported removal for all days and included allowing the monitor to charge, swimming, or medical examination. Completion of the tracking log was significantly associated with compliant accelerometer wear data at all time points (*P*<.001, Table 4).

**Table 4:** Prevalence of tracking log completion and manufacturer mobile app engagement with compliant accelerometry data collection among the active Lifestyle Intervention for oVarian cancer Enhanced Survival participants who agreed to wear an accelerometer at each study time point.

Time point^a,b^		Value, n (%)	*P* value
**Baseline (n=560)**			
	Tracking log	496 (88.6)	<.001
	Mobile app	198 (35.4)	.002
	Both	187 (33.4)	<.001
**6 months (n=394)**			
	Tracking log	327 (83.0)	<.001
	Mobile app	78 (19.8)	.06
	Both	60 (15.2)	.03
**12 months, n=260**			
	Tracking log	219 (84.2)	<.001
	Mobile app	41 (15.8)	.53
	Both	35 (13.5)	.16
**24 months (n=123)**			
	Tracking log	106 (86.2)	<.001
	Mobile app	13 (10.6)	.49
	Both	12 (9.8)	.50

^a^Percent values are calculated by dividing by the number of active participants who agreed to wear an accelerometer at each time point.

^b^Analyses were conducted using the Pearson chi-square tests.

At baseline, 35.4% (198/560) of the participants opted to use the mobile app; the average frequency of uploads during the 7-day wear period was 5 (median 6). At 6, 12, and 24 months, 19% (78/123), 15% (41/123), and 11% (13/123) of the participants repeated the use of the app, respectively. The average frequency of uploads at 6, 12, and 24 months was 5 (median 6), 6 (median 6), and 6 (median 6), respectively. The use of the mobile app was significantly associated with compliant data at baseline only (*P=*.002). Completion of both the tracking log and mobile app upload was significantly associated with compliant accelerometry data collected at baseline and 6 months (*P*=.001 and *P*=.03, respectively). Fewer participants completed both the tracking log and uploaded data through the mobile app at the following 12- and 24-month time points, with the majority of participants choosing to complete the tracking log only.

## Discussion

### Principal Findings

Given the current interest in objective measurement of physical activity levels in cancer survivors and reports of over- and under-reporting of physical activity using validated self-reported measures, a rigorous protocol, which includes SOPs, to successfully send, receive, collect, and download accelerometer data is recommended. A 2018 review of accelerometer-based activity monitoring in cancer survivors suggested that current efforts lack standardization in relation to the methodology used and further details of accelerometer data collection methods are lacking, making replication at best challenging [15]. To address this gap in current evidence, this study provides an adaptable, detailed protocol for consideration across trials, particularly those that are distance-delivered. On the basis of our findings, these protocols should include frequent communication via telephone or email, multiple methods of instruction for how to use the accelerometer (eg, video, paper, and telephone-based education), and a detailed mail tracking system. To increase the likelihood of receiving compliant and usable data, protocols should include having the participants upload their data in real time or record wear time on a standardized tracking form. Here, we report the study protocol, implementation, and initial compliant data for using accelerometers in a large, multisite national lifestyle intervention in a trial of ovarian cancer survivors. Data were collected from participants in 48 US states for objective measurement of activity. The LIvES research team has developed a reproducible protocol that can be used by others for the implementation of accelerometers in future research trials.

### Comparison With Prior Works

The majority (98%) of the data were collected within the predesignated windows for data collection. Agreement to wear the accelerometer did decrease over time among all protocol active participants. Our goal was to collect data from all active women within the subsample at all time points. However, a small percentage of the women were protocol noncompliant at previous time points or refused or were unable to wear the accelerometer (Table 1). Of those who provided verbal consent and availability to wear the accelerometer, 95.2% (533/560), 90.9% (358/394), 95.4% (248/260), and 97.6% provided compliant data at baseline, 6, 12, and 24 months, respectively. Our compliance rates for accelerometry were similar to large-scale prospective cohort studies in older women with single time point measurements [36] and were above the 71.6% wear time compliance estimates from adults wearing thigh and back-placed accelerometers [39] and much higher than estimates of 62.6% among children who participated in the National Health and Nutrition Examination Survey [40]. Compliant data on distance-delivered methodologies are sparse.

At the time of the development of this protocol, the GT9X was validated only for hip-worn measurements. Although the GT9X has since been validated for wristwear [18], it is unknown how this may have affected compliance in our sample. Among a large sample of European adults with a single time point of collection, 93.3% had valid data for wrist-worn accelerometers [41]. Comparatively, we demonstrated 95.2% valid compliant data from hip-worn accelerometers in our sample at baseline. The current literature suggests a poor correlation between wrist and hip-worn accelerometer counts per min; therefore, comparison of estimates from wear at the 2 different sites should be interpreted with caution [42,43]. To maintain consistency in the data collected, all participants were asked to wear GT9X on their nondominant hip at all time points. Furthermore, the hip placement of GT9X demonstrates better step count accuracy [18], which is an *a priori* behavior outcome for the trial. In addition, the quality of the data generated using the protocol will allow for time in sedentary bouts, light activity, and the development of population-specific cut-off points that accommodate the described reduction in peak oxygen consumption and relative intensity of activities among cancer survivors [44,45].

Contrary to other studies that may suggest that obese women have higher nonwear time of accelerometers [46], our results indicate that BMI did not have a significant effect on compliant data in ovarian cancer survivors. Tracking log completion remained strongly associated with compliant wear data across all time points. Calls at 6, 12, and 24 months became more streamlined and shorter in length, as many participants were already familiar with the protocol and felt comfortable with the device without additional telephone support at these time points. High compliance overall may reflect the older female sample of cancer survivors motivated to participate in a 24-month lifestyle intervention.

The use of the mobile app to upload data was optional, and 35.4% of women opted to use this app. We noted that age, though not statistically significant, influenced the use of the mobile app, suggesting that this approach may have higher adherence for younger cancer survivors. The mobile app allowed participants to upload data but they could not see their activity data to keep data collection blinded. This may have influenced the motivation for repeated app engagement. Over time, participants engaged less with the mobile app, results that are similar to the patterns previously reported in the literature for other health-related smartphone apps [47]. This warrants further studies, including new strategies to promote the continued use of technology. Of note, our instruction call notes from participants indicated that many participants did not find any benefit or did not feel like there was time available to complete both the tracking log and mobile app. An additional consideration in applying the findings of this research is our focus on a convenience sample of ovarian cancer survivors. Although we anticipate that the protocol will perform similarly in other cancer survivor populations, this is not a standard, particularly in relation to diversity in sex and education as well as cancer-related symptom burden and comorbidities.

### Future Directions

Beyond the scope of this protocol, but important to future research in this area, population-relevant cut-off points and algorithms need to be established for cancer survivors for both wrist and hip-worn accelerometers. The field of accelerometry is rapidly emerging, especially regarding standardized cut-off points. For data analysis related to objective physical activity data captured in this protocol, standard cut-off points as well as vector magnitude and total activity counts will be evaluated by the research team. The current literature suggests that vector magnitude may better discriminate between sedentary and light physical activity in women aged older than 60 years [48], although among breast cancer survivors, total activity counts may provide better estimates of moderate-to-vigorous physical activity [49]. These population-relevant cut-off points will allow for a more accurate interpretation of accelerometer data for the older female cancer survivor population; however, information remains limited for cut-off points specific to ovarian cancer survivors. Importantly, both age and the presence of comorbidities can influence accelerometer cut-off points [48], confounders that are highly relevant to this population.

In summary, we have developed a detailed protocol and related materials for collecting the accelerometry data from a large sample of cancer survivors who reside across the United States. This protocol has resulted in the acquisition of a robust data set for future analysis of physical activity in this population. This protocol and the related materials that were issued to participants are available through UACC-BMISR consultation services [50] in support of future research studies designed to capture repeated measures of activity in this vulnerable population.

## Abbreviations

APAQ: Arizona Physical Activity Questionnaire
BMISR: Behavioral Measurement and Interventions Shared Resource
HIPAA: Health Insurance Portability and Accountability Act
LIvES: Lifestyle Intervention for oVarian cancer Enhanced Survival
OR: odds ratio
SOP: standard operating procedure
UACC: University of Arizona Cancer Center
USPS: United States Postal Service
